# Notes on the green lacewing subgenus Ankylopteryx Brauer, 1864 (*s. str.*) (Neuroptera, Chrysopidae) from China, with description of a new species

**DOI:** 10.3897/zookeys.906.46438

**Published:** 2020-01-22

**Authors:** Yunlong Ma, Xingke Yang, Xingyue Liu

**Affiliations:** 1 Department of Entomology, China Agriculture University, Beijing 100193, China China Agriculture University Beijing China; 2 Key laboratory of Zoological Systematics and Evolution, Institute of Zoology, China Academy of Sciences, Beijing 100101, China Institute of Zoology, China Academy of Sciences Beijing China

**Keywords:** Description, new species, synonym, key

## Abstract

A taxonomic study of the green lacewing subgenus Ankylopteryx Brauer, 1864, from China is presented. Eight species of this subgenus are recorded from China. A new species, namely Ankylopteryx (A.) yangi**sp. nov.**, is described. Ankylopteryx (A.) delicatula Banks, 1937, and Ankylopteryx (A.) ferruginea Tsukaguchi, 1995 are recorded from China for the first time. Four new junior synonyms are proposed for Ankylopteryx (A.) octopunctata candida Fabricius, 1798: i.e. Ankylopteryx (A.) fraterna Banks, 1939, Ankylopteryx (A.) laticosta Banks, 1939, Ankylopteryx (A.) lii Yang, 1987, and Ankylopteryx (A.) tibetana Yang, 1987. A revised key to the Chinese species of the subgenus Ankylopteryx is provided.

## Introduction

The green lacewing genus *Ankylopteryx* (Chrysopidae, Chrysopinae, Ankylopterygini) was established by Brauer (1864), with *Chrysopa venusta* Hagen, 1864 as its type species by subsequent designation by Tjeder (1966). This genus is characterized by the strongly broadened, usually immaculate basal part of the forewing costal space and the presence of the pseudopenis in the male genitalia. This genus consists of two subgenera, i.e. *Ankylopteryx* (*s. str.*) Brauer and *Sencera* Navás (Brooks and Barnard 1990). The subgenus Sencera, which occurs in the Oriental and Australian regions (Breitkreuz et al. 2015), was firstly established as a genus by Navás (1925) and subsequently treated as a subgenus of *Ankylopteryx* (*s. l.*) by Brooks and Barnard (1990) on account of striking similarity on external and genital characters proposed by Brooks (1983). *Sencera* differs from *Ankylopteryx* (*s. str.*) by the absence of the forewing intramedian cell (Brooks and Barnard 1990). However, *Sencera* was synonymized with *Ankylopteryx* (*s. str.*) by Tsukaguchi (1995), but it was still treated as a valid subgenus by New (2003), Yang et al. (2005), and Breitkreuz et al. (2015). Breitkreuz et al. (2015) revised the subgenus Sencera but questioned its subgeneric status. The subgenus Ankylopteryx (*s. str.*), containing 44 described species, is relatively poorly studied compared to *Sencera*. Previously, 10 species of *Ankylopteryx* (*s. str.*) were recorded from China (Yang et al. 2005). Recently, we examined about 100 specimens of *Ankylopteryx* (*s. str.*) from China, including several type specimens of the Chinese species described by Chikun Yang (i.e. *A. lii* Yang, 1987, *A. magnimaculata* Yang, 1987, and *A. tibetana* Yang, 1987). Accordingly, we present an overview of the species of *Ankylopteryx* (*s. str.*) from China and describe a new species. *Ankylopteryx* (*s. str.*) *delicatula* Banks, 1937 and *A. ferruginea* Tsukaguchi, 1995 are newly recorded from China. A revised key to the Chinese species of *Ankylopteryx* (*s. str.*) after Yang et al. (2005) is provided.

## Material and methods

Terminology of wing venations in Neuroptera was proposed in a number of studies, such as Tillyard (1916), Comstock (1918), Adams (1967), Kukalová-Peck (1991), Kukalová-Peck and Lawrence (2004), and Breitkreuz et al. (2017), but with different interpretations on certain veins. The terminology of wing venation used in this paper mainly follows the previous studies on green lacewings, e.g. Tillyard (1916), Tauber (2003), and Tauber et al. (2017). Terminology of genitalia in Neuroptera was comprehensively studied by Acker (1960) and subsequently modified in a series of works (e.g. Tjeder 1966, 1970; Adams 1969; Principi 1977; Aspöck 2002; Aspöck and Aspöck 2008). In particular, Aspöck and Aspöck (2008) provided homology interpretation on the genital segments 8–11 based on the gonocoxite concept. Nevertheless, the terminology of genitalia used in this paper still follows some major works on systematics of green lacewings (e.g. Tjeder 1970; Principi 1977; Adams and Penny 1985; Brooks and Barnard 1990; Tauber 2003; Tauber et al. 2017).

Measurement of head width was made across the widest part of the dorsum of head including the compound eyes; the ratio of head width : eye width used the distance between middle of vertex and the maximum width of the compound eye; prothoracic length and width was respectively measured along the dorsal midline and at the widest position (straight line distance across the posterior margin) of prothorax; the wing length and width was respectively measured at the longest and widest portion of wing. The genitalia were macerated in 10% KOH, then washed twice in dH_2_O and stained with Chlorazol Black in 80% ethanol. The dissected genitalia from dried specimens were placed in glycerine in a tube pinned beneath the specimen. The genitalia from specimens preserved in alcohol were placed in 95% ethanol in a tube, placed with the remaining part of specimen in a larger tube filled with 95% ethanol.

Specimens herein examined are deposited in the Entomological Museum of China Agricultural University (CAU), Beijing except for the type of *Ankylopteryx doleshalii* Brauer, 1864. Other collections with primary types of relevant species cited in this paper are listed below.

**CLMX** Collection of Liang Minxuan, Hong Kong, China

**MCZ** Museum of Comparative Zoology, Harvard University, Cambridge, Massachusetts, USA

**MNHN** Muséum National d’Histoire Naturelle, Paris, France

**NHMV** Naturhistorisches Museum, Wien, Austria

**NSMT** National Science Museum (Natural History), Tokyo, Japan

**UOP** Osaka Prefecture University, Osaka, Japan

**ZMUK** Universität Kiel, Zoologisches Museum, Kiel, Germany

## Taxonomy

### Subgenus Ankylopteryx

Taxon classificationAnimaliaNeuropteraChrysopidae

Brauer, 1864

41974DFA-BF14-5E37-8DFF-AA488DDE069C

Ankylopteryx : Brauer 1864: 899; Hagen 1866: 377; Kuwayama 1924: 7; Banks 1938: 225; Tjeder 1966: 497; Hölzel 1970: 50; Hölzel 1973: 382; New 1980: 15; Brooks 1983: 6; Tsukaguchi 1985: 505; Brooks and Barnard 1990: 125, 155; Tsukaguchi 1995: 122; Yang et al. 2005: 49.

#### Type species.

*Chrysopa venusta* Hagen, 1853, by subsequent designation by Tjeder (1966).

#### Synonym.

*Ethiochrysa*Fraser 1952: 57; Brooks and Barnard 1990: 155 (synonymized *Ethiochrysa* Fraser, 1952 with *Ankylopteryx* Brauer, 1864). Type species: *Ethiochrysa polychlora* Fraser, 1952, by monotypy.

#### Diagnosis

(adapted from Brooks and Barnard 1990). Small to medium-sized green lacewings, body generally pale green. Head narrow (head width : eye width = 1.9–2.6 : 1), marked with black or red stripes on clypeus, gena or frons; maxillary palp and labial palp narrow, elongate apically; antenna nearly as long as forewing. Pronotum narrow, sometimes marked with black lateral spot, and with pale long fine setae; meso- and metanotum sometimes with broad black markings. Legs with protibia and mesotibia often marked with spots at median portion; metatibia seldom marked. Forewing broad (length : width = 2.1–2.5 : 1); marked with large black or brown spots or suffusion; costal space broad near wing base; costal vein with erect long setae; Sc very short; pterostigma often with black spots; Subcosta (Sc) and Radius (R) closely spaced; first intramedian cell present; two gradate series of crossveins present, slightly divergent anteriad, basal inner gradate series meeting Psm; veins not crassate in male. Hind wing narrow (length : width = 3.0–4.0 : 1). Abdomen with sparse, long setae, with terga often marked; callus cerci ovoid; both male and female ectoprocts fused dorsally with slight dorsal invagination; male sterna 8+9 fused, microtholi absent. Female sternum 7 posteriorly truncate in ventral view with small setose apical tubercle.

**Distribution.** Afrotropical, Australian, and Oriental regions.

### Ankylopteryx (A.) delicatula

Taxon classificationAnimaliaNeuropteraChrysopidae

Banks, 1937

87E9D396-B277-582C-984D-48AF0E58ABC7

Figs 1 , 10–14 , 15–17 , 112

Ankylopteryx (A.) delicatula : Banks 1937: 280 (original: Ankylopteryx; type locality: Ryukyu (Japan, Okinawa); syntypes in MCZ); Kuwayama 1964: 42 (Ankylopteryx); Brooks and Barnard 1990: 265 (Ankylopteryx (Ankylopteryx)); Tsukaguchi 1995: 123 (key to Japanese species), 126, fig. 100 (Ankylopteryx).

#### Material examined.

**China**: 1 ex, Yunnan, Jinghong, Sanchahe, 620 m, 1981.IV.12, Yang Chikun (CAU); 1 ♀, Yunnan, Yingjiang, Tongbiguan, Jinzhuzhai, 2012.V.2, Liang Feiyang (CAU); 1 ♂, Hong Kong, 2015.V, Liang Minxuan (CLMX).

#### Diagnosis.

Frons with two brownish small spots between antennae, two brownish stripes below toruli; frontal markings more or less curved posteriorly and contiguous with clypeal markings anteriorly; gena with a long brownish stripe. Protibia and mesotibia with median markings. Both wings distinctly marked with brownish vittae along posterior margins near base and medial fork to apex of distal cubital cell (*dcc*). Abdomen with brownish markings on terga 2–8.

#### Supplemental description.

Female: Sternum 8 distinctly convex at median part of posterior margin, with setae apically. Subgenitale stubby, bilobed apically; spermatheca round, as wide as long; spermaduct coiled, about three times as long as spermatheca.

#### Distribution.

China (Yunnan, Hong Kong); Japan (Okinawa).

#### Remarks.

The frontal spots between antennae and vittae on both wings in our examined specimens from Yunnan are same with that in the type of *A. delicatula*, as originally described. Accordingly, we identified the above two specimens from Yunnan to be *A. delicatula*, which is newly recorded from China.

### Ankylopteryx (A.) doleschalii

Taxon classificationAnimaliaNeuropteraChrysopidae

Brauer, 1864

76108521-A6F2-5059-A306-34F29A0BF8B3

Figs 2 , 18–22 , 23–29 , 112

Ankylopteryx (A.) doleschalii : Brauer 1864: 901 (original: Ankylopteryx; type locality: “Amboina” [Ambonia] (Indonesia, Maluku Prov.); holotype in NHMV); Brauer 1866: 37 (Ankylopteryx); van der Weele 1909: 60 (Ankylopteryx); Banks 1937: 280 (Ankylopteryx); Banks 1939: 473 (key to Chinese species); Kuwayama 1964: 42 (Ankylopteryx); Brooks and Barnard 1990: 265 (Ankylopteryx (Ankylopteryx)); New 2003: 163 (Ankylopteryx (Ankylopteryx)); Yang et al. 2005: 51 (Ankylopteryx (Ankylopteryx)).

#### Material examined.

Holotype ex, Indonesia, Amboina, 1950 (NHMV). Paratype 1 ♂, same data as holotype (NHMV).

#### Diagnosis.

Stripes below toruli absent; frontal markings not curved posteriorly, anteriorly contiguous with clypeal markings and genal markings. Protibia and mesotibia with median markings. Both wings with brownish marking patterns. First intramedian cell very long and narrow.

#### Distribution.

China (Hainan); Indonesia (Maluku).

#### Remarks.

This species was recorded from Hainan by Banks (1937). Unfortunately, we have not examined any specimen of this species from Hainan. Considering the greatly disjunct distribution records of this species (i.e. Ambonia and Hainan), there is a possibility that *A. doleschalii*, from Hainan was a misidentification of *A. gracilis* (a morphologically similar species widely distributed in eastern and southeastern Asia).

### Ankylopteryx (A.) ferruginea

Taxon classificationAnimaliaNeuropteraChrysopidae

Tsukaguchi, 1995

8AA1793B-5AC2-5F7A-910E-0E4BCF067889

Figs 3 , 30–32 , 33–35 , 106 , 110 , 112

Ankylopteryx (A.) ferruginea : Tsukaguchi 1995: 127 (original: Ankylopteryx; type locality: Iriomote (Japan, Okinawa); holotype in UOP).

#### Material examined.

**China**: 1 ♂, Guangxi, Longzhou, Nonggang, 240 m, 1982.V.19, Li Fasheng (CAU); 1 ♂, Guangxi, Longzhou, Nonggang, 240 m, 1982.V.20, Yang Chikun (CAU); 1 ♂, 1 ♀, Guangxi, Longzhou, Nonggang, 240 m, 1982.V.21, Li Fasheng (CAU); 1 ♀, Guangxi, Ningming, Longrui, 180 m, 1984.V.16, Li Fasheng (CAU); 1 ♀, Guangxi, Ningming, Longrui, 180 m, 1984.V.17, Li Fasheng (CAU); 1 ♀, Yunnan, Jinghong, Yexianggu, 2015.IV.17, Liang Feiyang (CAU); 1 ♂, Yunnan, Lancang, Yunxian, Xingtu, 2017.VII. 20, Yang Mengxian (CAU); 1♀, Hainan, Yinggeling, Wang Jianyun (CAU). **Japan**: 2 ♂, Okinawa, Iriomote-jima, Airagawa, Komi, 2012.VI.2, Liu Xingyue (CAU).

#### Diagnosis.

Stripes below toruli absent; clypeal markings indistinct or absent, if present, contiguous with indistinct genal markings. Pro-, meso-, metibia with median markings. Both wings with yellowish marking patterns. First intramedian cell very long and narrow. General width of gonarcus narrow; entoprocessus attached at basal fourth of gonarcus, about 3/4 times as long as gonarcus; pseudopenis long and straight, about two times as long as entoprocessus.

#### Distribution.

China (Guangxi, Yunnan); Japan (Okinawa).

#### Remarks.

This species is newly recorded from China. Tsukaguchi (1995) stated that two forewing m-cu crossveins are present before first intramedian cell. However, this character is variable among individuals of this species based on the specimens we examined. Other external diagnostic characters and genital characters in the Chinese specimens fit well with the original description, which confirms our identification of this species.

This species is similar to *A. gracilis*, based on the long and narrow first intramedian cell, but differs from the latter two species by the absence of markings on frons between antennae (present in *A. doleschalii*, and *A. gracilis*), the yellowish wing marking pattern (wing marking pattern much darker in *A. doleschalii*, and *A. gracilis*) and the narrow general width of gonarcus (strongly broad in *A. gracilis*).

### Ankylopteryx (A.) gracilis

Taxon classificationAnimaliaNeuropteraChrysopidae

Nakahara, 1955

D15ED022-B760-5130-A72A-47B2414AAF7E

Figs 4 , 36–40 , 41–43 , 44–47 , 107 , 111 , 113

Ankylopteryx (A.) gracilis : Nakahara 1955: 143, pl. 21 fig. 1 (original: Ankylopteryx; type locality: “Formosa” [Taiwan] (China); holotype in NSMT); Kuwayama 1964: 42 (Ankylopteryx); Tsukaguchi 1985: 505 (Ankylopteryx); Brooks and Barnard 1990: 265 (Ankylopteryx (Ankylopteryx)); Tsukaguchi 1995: 123 (key to Japanese species and a key to the third instar larvae), 129 (Ankylopteryx); New 2003: 164 (Ankylopteryx (Ankylopteryx)); Yang et al. 2005: 51 (key to Chinese species, Ankylopteryx (Ankylopteryx)).

#### Material examined.

**China**: 1 ♀, Guangxi, Longzhou, Nonggang, 240 m, 1982.V.19, Yang Chikun (CAU); 1 ♂, 1 ex, Guangxi, Longzhou, Nonggang, 240 m, 1982.V.20, Yang Chikun (CAU); 1 ♂, 2 ♀, Guangxi, Longzhou, Nonggang, 240 m, 1982.V.20, Li Fasheng (CAU); 13 ♂, 2 ♀, Yunnan, Jinghong, Sanchahe, 620 m,1981.IV.12, Yang Chikun (CAU); 1ex, Hainan, Baisha, Hongmao, 430 m, 2007.V.18, Liu Xingyue (CAU); 1 ♀, Hainan, Baisha, Yinggeling, Hongxin, 2007.V.23-24, Liu Jingxian (CAU); 2♂, Taiwan, Nantou, Yüchih, Lienhuachih, 675 m, 2010.XI.11, Yang Ding (CAU). **Japan**: 3♂, Okinawa, Iriomote-jima, Airagawa, Komi, 2012.VI.29, Liu Xingyue (CAU). **Vietnam**: 1 ♀, Quang Nam, Phuoc Son, Phue My, 580 m, 2012.V.6, Liu Xingyue (CAU). **Laos**: 1 ♀, Vientiane Prov., Phou Panang NBCA, 260 m, 2016.III.27, Liu Xingyue (CAU).

#### Diagnosis.

Frons with three spots between antennae, stripes below toruli absent; frontal markings not curved posteriorly contiguous with clypeal markings anteriorly; gena with long brownish stripe. First intramedian cell very long and narrow, general width of gonarcus very broad; entoprocessus attached at about median part of gonarcus, about half as long as gonarcus; pseudopenis long and straight, about twice as long as entoprocessus.

#### Distribution.

China (Guangxi, Yunnan, Hainan, Taiwan); Japan (Okinawa); Vietnam (Quang Nam); Laos (Vientiane).

#### Remarks.

The frontal spots between antennae, the long and narrow intramedian cell, and the genital characters assigned the specimens examined to *A. gracilis*.

In the original description of *A. gracilis*, Nakahara (1955) stated that the legs of this species are immaculate, and this feature was followed by Tsukaguchi (1995) as a diagnostic character of this species. However, among the specimens examined here, we found that the markings on foretibia and mesotibia are either absent or present. After examination of the male genitalia of these specimens, no significant differences among them could be found, indicating that they belong to a same species. Therefore, we consider the presence/absence of markings on tibia to be intraspecific variation, which is common in some other species of this subgenus.

### Ankylopteryx (A.) magnimaculata

Taxon classificationAnimaliaNeuropteraChrysopidae

Yang, 1987

3EAD8194-F65C-5647-85F7-2881954DF7A7

Figs 6 , 58–63 , 64–66 , 102 , 112

Ankylopteryx (A.) magnimaculata : Yang 1987: 204 (original: Ankylopteryx; type locality: Dongjiong (China, Xizang, ä); holotype in CAU); Yang et al. 2005: 51 (key to Chinese species), 54 (Ankylopteryx (Ankylopteryx)).

#### Material examined.

Holotype ♂, Xizang, Zäyu, Dongjiong, 1570 m, 1978.VI.26, Li Fasheng (CAU).

#### Diagnosis.

Two brownish vittae on frons, clypeus, and labrum; gena with long brownish stripes contiguous with above vitta. Protibia and mesotibia with median markings. Forewing with pterostigma brown, extending over four crossveins; large brownish vittae present along posterior margins at basal third, enclosing over five veins on wing margin; first intramedian cell short and wide. General width of gonarcus narrow; entoprocessus attached near base of gonarcus, slightly longer than gonarcus; pseudopenis short and curved, about 1.5 times as long as entoprocessus.

#### Distribution.

China (Xizang).

#### Remarks.

This species can be distinguished from all the other species of *Ankylopteryx* (*s. str.*) from China by the large brownish vittae along posterior margin at basal third of both wings.

### Ankylopteryx (A.) octopunctatacandida

Taxon classificationAnimaliaNeuropteraChrysopidae

(Fabricius, 1798)

6CFF5461-747E-56FD-9433-66D5D65A267A

Figs 5 , 7 , 8 , 58–63 , 64–66 , 67–72 , 73–76 , 77–82 , 83–85 , 99–101 , 108 , 113

Ankylopteryx (A.) octopunctata candida : Fabricius 1798: 202 (original: Hemerobius; type locality: “India orientali” [E. India]; holotype in ZMUK); Schneider 1851: 161 (Chrysopa); Walker 1853: 274 (Chrysopa); Brauer 1864: 900 (Ankylopteryx); van der Weele 1909: 58 (Ankylopteryx); Banks 1939: 473 (key to Chinese species, Ankylopteryx); Brooks and Barnard 1990: 265 (Ankylopteryx (Ankylopteryx)).
Ankylopteryx (A.) fraterna Banks, 1939: 473 (key to Chinese species; original: Ankylopteryx; type locality: Guangdong and Hainan (China); syntypes in MCZ); Yang et al. 2005: 51 (A key to Chinese species, Ankylopteryx (Ankylopteryx)). **syn. nov.**
Ankylopteryx (A.) laticosta Banks, 1939: 472 (original: Ankylopteryx; type locality: Guangdong and Hainan (China); syntypes in MCZ), 473 (key to Chinese species); Brooks and Barnard 1990: 265 (Ankylopteryx (Ankylopteryx)); Yang et al. 2005: 51 (key to Chinese species), 53 (Ankylopteryx (Ankylopteryx)). **syn. nov.**
Ankylopteryx (A.) lii Yang 1987: 204 (original: Ankylopteryx; type locality: Shajiong (China, Xizang, Zäyu); holotype in CAU); Yang et al. 2005: 51 (key to Chinese species), 54 (Ankylopteryx (Ankylopteryx)). **syn. nov.**
Ankylopteryx (A.) tibetana Yang 1987: 205 (original: Ankylopteryx; type locality: Dongjiong (China, Xizang, Zäyu); holotype in CAU); Yang et al. 2005: 51 (key to Chinese species), 56 (Ankylopteryx (Ankylopteryx)). **syn. nov.**

#### Material examined.

**China**: 1 ♂, Fujian, Shaowu, 1943.IX.1 (CAU); 1 ♂, Fujian, Dehua, Shuikou, 1974.XI.6, Li Fasheng (CAU); 3 ♂, 1 ♀, Fujian, Dehua, Shuikou, 1974.XI.6, Yang Chikun (CAU); 1 ♂, Fujian, Dehua, Shuikou, 1974.XI.13, Yang Chikun (CAU); 1♀, Jiangxi, Shangrao, 1978.IV.30, Yang Chikun; 1 ♂, Guangxi, Longzhou, Nonggang, 240 m, 1982.V.18, Yang Chikun (CAU); 1 ♂, Guangxi, Longzhou, Nonggang, 240 m, 1982.V.19, Li Fasheng (CAU); 2 ♂, Guangxi, Longzhou, Nonggang, 240 m, 1982.V.18, Li Fasheng (CAU); 1ex, Guangxi, Ningming, Longrui, 180 m, 1984.V.16, Li Fasheng (CAU); 2 ♀, Guangxi, Ningming, Longrui, 180 m, 1984.V.17, Li Fasheng (CAU); 1 ♂, Guangxi, Jinxiu, 720 m, 1982.VI.11, Li Fasheng (CAU); 3 ♀, Sichuan, Leshan, 1978.IX.20, Li Fasheng (CAU); 1 ♂, Chongqing, Liangping, Luojia, Shapingba, Li Zhifei (CAU); 1 ♀, Guizhou, Libo, Maolan, Banzhai, 2013.X.13, Liu Xingyue (CAU); 1 ♂, 1 ♀, Guizhou, Libo, Maolan, Limingguan, 2013.X.14, Liu Xingyue (CAU); 1 ♂, 1 ♀, Guizhou, Libo, Maolan, Limingguan, 2013.X.14, Liang Feiyang (CAU); 2 ♀, Yunnan, Puer, Taiyanghe National Forestry Park, 1450 m, E101.3 N22.68, 2016.VIII.12, Jiang Yunlan (CAU); 1 ♀, Guangdong, Huizhou, Xiangtoushan, Wang Mengqing (CAU); 1 ♂, 1 ♀, Guangdong, Zhaoqing, Dinghushan Nature Reserve, Lyu Yanan (CAU); 1 ♂, Hainan, Diaoluoshan, 2014.V.4, Lu Xiumei (CAU); 1 ♂, Xizang, Zäyu, Shajiong, 1570 m, 1978.VI.26, Li Fasheng (holotype of *Ankylopteryx lii* Yang, 1987) (CAU); 1 ♂, Xizang, Zäyu, Dongjiong, 1570 m, 1978.VI.26, Li Fasheng (holotype of *Ankylopteryx tibetana* Yang, 1987) (CAU); 1 ♂, Xizang, Zäyu, Shajiong, 1700 m, 1978.VI.25, Li Fasheng (paratype of *Ankylopteryx tibetana* Yang, 1987) (CAU); 1 ♂, 5 ♀, Taiwan, Pingtung, Lilungshan, 2013.VI.18, Liang Feiyang (CAU). **Japan**: 5 ♀, Okinawa, Iriomote-jima, Airagawa, Komi, 2012.VI.29, Liu Xingyue (CAU). **Laos**: 1 ♂, 2 ♀, Luang Namtha, Nam Ha NBCA, Along Route 3, 690–750 m, 2016.III.22, Liu Xingyue (CAU).

#### Diagnosis.

Stripes below toruli from absent to visibly present; frontal markings contiguous with clypeal markings and genal marking. Scape usually with brownish stripe. First intramedian cell short and wide. General width of gonarcus normal; entoprocessus attached at basal fifth to fourth of gonarcus, about as long as gonarcus; pseudopenis short and curved, about twice as long as entoprocessus.

#### Distribution.

China (Fujiang, Guangxi, Sichuan, Chongqing, Guizhou, Yunnan, Guangdong, Hainan, Xizang); Japan (Okinawa); Laos (Luang Namtha); India (eastern India).

#### Remarks.

Seven subspecies hitherto are placed under the species *A. octopunctata*, which are separately distributed. According to the known distribution of *A. octopunctata candida* and the character mentioned above, we assigned the materials examined to this subspecies.

#### Synonyms.

*Ankylopteryx fraterna* and *A. laticosta* were first recorded by Banks (1939). In the original literature, he mentioned that the key difference between *A. fraterna* and *A. octopunctata candida* was the presence or absence of brownish stripe on scape (absence in *A. fraterna* versus presence in *A. octopunctata candida*); differences between *A. laticosta* and *A. octopunctata candida* are the coloration of the costal vein (black for a short distance in *A. laticosta* versus not black in *A. octopunctata candida*), costal area (unusually broad in *A. laticosta* versus normal in *A. octopunctata candida*), and the number of lower Banksian cells (two in *A. laticosta* versus three in *A. octopunctata candida*). We have studied the specimens of *A. octopunctata candida* from various localities and found the difference mentioned above is continuous in this species. We, therefore, synonymize *A. fraterna* and *A. laticosta* with *A. octopunctata candida*. We have also studied the holotypes of *A. lii* and *A. tibetana*. The external characters (frontal stripes and markings, and markings on both wings) of three nominal species are similar. We dissected specimens of each of the three species, found no significant difference among them, and therefore confirmed the synonymization of *A. lii* and *A. tibetana* with *A. octopunctata candida*.

### Ankylopteryx (A.) quadrimaculata

Taxon classificationAnimaliaNeuropteraChrysopidae

(Guérin-Méneville, 1844)

5D9A50D1-244B-5EDF-82F1-C96307EE228F

Ankylopteryx (A.) quadrimaculata : Guérin-Méneville 1844: 388 (original: Hemerobius; type locality: “Chine” [China]; type in MNHN); Schneider 1851: 162 (Chrysopa); Hagen 1866: 380 (Ankylopteryx); Brooks and Barnard 1990: 265 (Ankylopteryx (Ankylopteryx)); Yang et al. 2005: 56 (Ankylopteryx (Ankylopteryx)).

#### Distribution.

China.

#### Remarks.

The original description of this species (Guérin-Méneville 1844) is too simple, and the only informative descriptions are the body length (= 12 mm), forewing span (= 36 mm), and the presence of stripes below toruli. Nevertheless, the stripes below toruli are also present in *Ankylopteryx octopunctata* (Fabricius, 1793), *A. tesselata* Needham, 1909, *A. nonelli* Navás, 1913, *A. nepalensis* Hölzel, 1973, and *A. yangi***sp. nov.** Thus, the validity of *A. quadrimaculata* is doubtful and this species may be a synonym of those species mentioned above except *A. yangi***sp. nov.** (differences between *A. yangi***sp. nov.**, and *A. quadrimaculata* are outlined below in the Remarks for *A. yangi***sp. nov.**). This species is not included in the present key, but still treated as a valid species until the type is examined.

### Ankylopteryx (A.) yangi sp. nov.

Taxon classificationAnimaliaNeuropteraChrysopidae

73095F4D-A45B-5F21-B355-51606C3164D8

http://zoobank.org/5E1748C9-4C93-4DAC-9C9D-66C63FAA8EC8

Figs 9 , 86–90 , 91–94 , 95–98 , 103 , 105 , 112

#### Material examined.

Holotype ♂, China, Guizhou, Libo, Maolan, Limingguan, 2013.X.14, Liu Xingyue (CAU). Paratypes 1 ♀, China, Guizhou, Libo, Maolan Limingguan, 2013.X.14, Liang Feiyang (CAU); 1 ♀, Taiwan, Pingtung, Lilungshan, 2013.VI.18, Liang Feiyang (CAU).

#### Diagnosis.

Frons with three brownish small spots between antennae, and anteriorly with two arcuate markings, which are more or less connected with each other at posterior ends; gena with long brownish stripe; clypeal markings contiguous with frontal markings posteriorly and labial markings anteriorly; maxillary palp and labial palp pale green. Thorax with mesonotum entirely brown; protibia and mesotibia with median markings. Forewing with intramedian cell short and wide. Abdomen with brownish markings on terga 2–8.

#### Description.

Measurements: Head width 0.7–1.0 mm; ratio of head width/eye width 1.72–1.74; prothorax 0.7–0.9 mm long and 0.85–1.0 mm wide. Forewing 11.5–13.9 mm long, 5.0–6.0 mm wide; length of first intramedian cell 0.98 mm; 12 radial cells; 4–5 Banksian cells (b cells), 4–5 lower Banksian cells (b' cells); 7–8 inner gradates, 7–10 outer gradates. Hind wing 10.0–13.5 mm long, 3.2–4.0 mm wide; 11 radial cells; 4–6 Banksian cells (b cell), 5–6 lower Banksian cell (b' cells); 4–6 inner gradates, 5–7 outer gradates.

Male. Head with vertex creamy yellow, immaculate; frons creamy yellow, with three small spots between antennae, brownish stripes below toruli, and a pair of median arcuate markings anteriorly, more or less connected with each other posteriorly; gena with long brownish stripe extending along inner ocular margin to posterolateral part of clypeus; tentorial pits with brownish margins; scape with brownish stripe; clypeus with brownish arcuate markings contiguous with frontal markings anteriorly and labial markings posteriorly; maxillary palp and labial palp pale green.

Prothorax almost pale green, with wide, brownish lateral stripe, and with white long setae. Mesothorax entirely brown dorsally, with sparse white long setae. Metathorax pale green, with sparse white long setae. Legs pale green, tarsomere 5 and pretarsal claws dark brown; protibia with a brownish median marking; mesotibia with a smaller median brownish marking.

Forewing broad, slightly tapering apically, hyaline; pterostigma brownish, extending over four crossveins; setae almost whitish; veins mostly pale green; costal crossveins at junctions with wing margin, radial crossveins at junctions with R and dcc brown; Radical sector (Rs) sinuated; transverse veins pale green; gradate series of crossveins almost brown; dcc closed. Hind wing narrow, more acutely tapering apically than forewing, hyaline; pterostigma faint, extending over three crossveins; transverse veins pale green; gradate series of crossveins almost brown.

Abdomen pale green, with brownish markings on terga 2–8, and tergal markings slightly wider than that on anteriorly neighbouring terga. Abdominal setae white, microsetae dense, and long setae sparse.

T9+ectoproct about half as long as tergum 8, with dorsal invagination shallow; ectoproct with rounded dorsal and posterior margins; callus cerci rounded, trichobothria densely ranged. S8+9 fused, as long as wide, with line of fusion not demarcated; lateral margin almost straight, posterior margin rounded. Only gonarcus, entoprocessus, and pseudopenis present. Gonarcus broadened at apex of lateral arms. Gonarcus with the general width normally broad; entoprocessus attached at about basal fourth of gonarcus, about half as long as gonarcus, medially fused forming an arch over pseudopenis; pseudopenis about twice as long as entoprocessus, broadened subapically, long and distinctly curved, and pointed apically; gonosaccus with sparse setae.

Female. External characters same as male. Sternum 7 distinctly convex posteromedially, setose posteriorly. Subgenitale and spermatheca with spermaduct present; subgenitale bilobed apically; spermatheca round, as wide as long; spermaduct coiled, much longer than spermatheca.

#### Distribution.

China (Guizhou, Taiwan).

#### Etymology.

This new species is dedicated to Professor Yang Chikun, who made tremendous contributions to the taxonomy of Chrysopidae from China.

#### Remarks.

This new species appears to be closely related to *A. octopunctata candida* in having similar frontal markings, but it differs from the latter species by the presence of median arcuate frontal markings (absent in *A. octopunctata candida*), the ratio of gonarcus/entoprocessus (2.0 in *A. yangi* versus 1.0 in *A. octopunctata candida*), and the distinctly curved pseudopenis (moderately curved in *A. octopunctata candida*).

##### Key to Chinese species of *Ankylopteryx* (*s. str.*) (revised after Yang et al. 2005)^1^

**Table:** 

1	First intramedian cell very long and narrow	**2**
–	First intramedian cell short and wide	**4**
2	Frontal area between antennae with three black spots (Fig. 36); scape and pedicel with brownish stripes (Fig. 37)	***A. gracilis* Nakahara, 1955**
–	Frontal area between antennae immaculate; scape and pedicel immaculate	3
3	Both wings with yellowish marking patterns (Figs 28, 29)	***A. ferruginea* Tsukaguchi, 1995**
–	Both wings with brownish marking patterns (Figs 21, 22)	***A. doleschalii* Brauer, 1864**
4	Frontal area between antennae with brownish spots	**5**
–	Frontal area between antennae immaculate	**6**
5	Three spots present (Fig. 86); both wings without large brownish vittae along posterior margins (Fig. 90)	***A. yangi* sp. nov.**
–	Two spots present (Fig. 10); both wings with large brownish vittae along posterior margins near base (Fig. 14)	***A. delicatula* Banks, 1937**
6	Both wings with brownish vittae along posterior margins (Figs 62, 63); general width of gonarcus narrow (Figs 65, 66, 102)	***A. magnimaculata* Yang, 1987**
–	Both wings without brownish vittae along posterior margins (Figs 71, 72); general width of gonarcus normal (Figs 75, 76, 100)	***A. octopunctata candida* (Fabricius, 1798)**

## Plates

**Figures 1–9.**
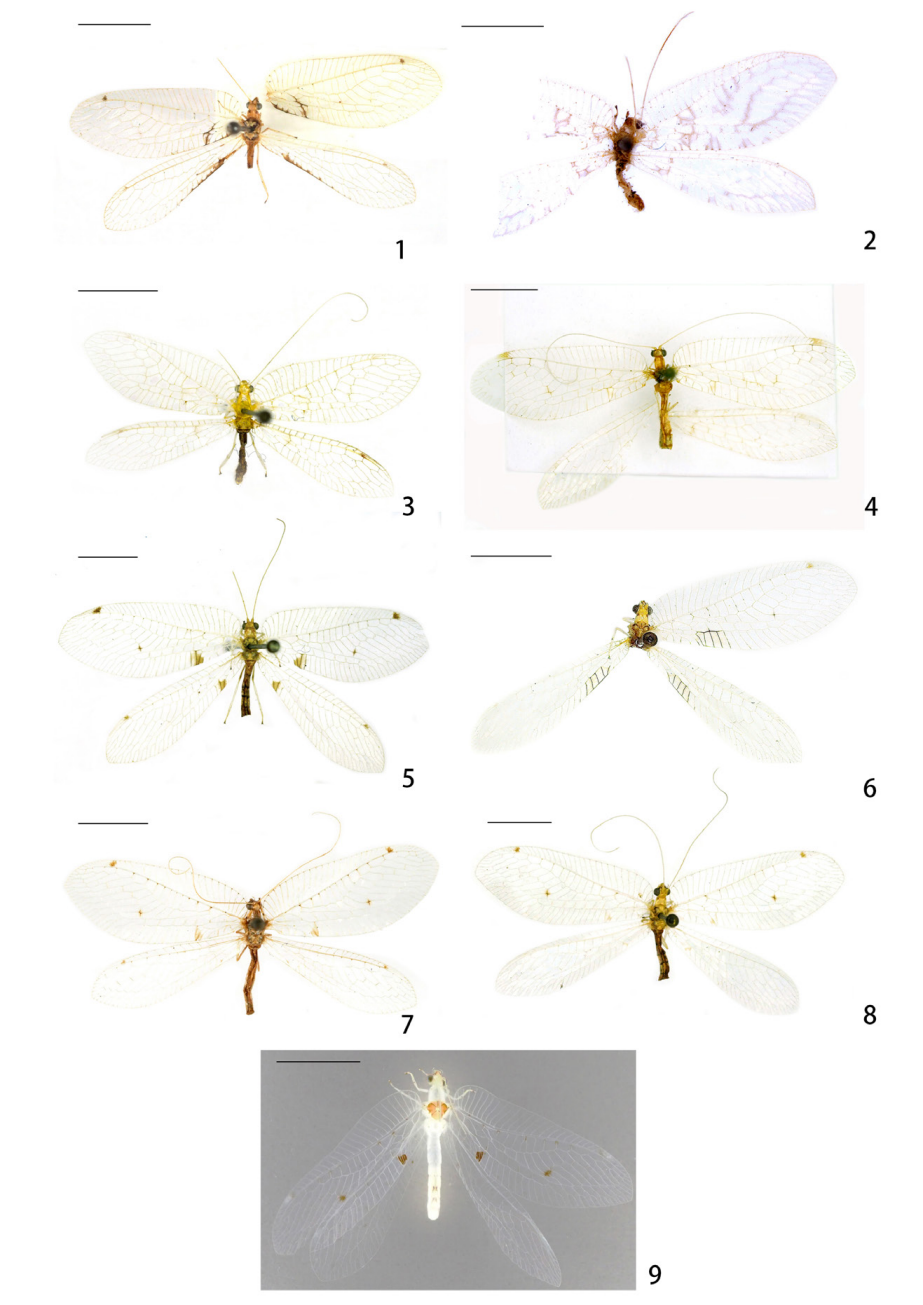
Habitus photos of the *Ankylopteryx* species. **1**Ankylopteryx (A.) delicatula Banks, 1937 (Yunnan, Jinghong, sex unknown, CAU) **2**Ankylopteryx (A.) doleschalii Brauer, 1864, paratype (Indonesia, Ambonia, paratype, male, provided by NHMV) **3**Ankylopteryx (A.) ferruginea Tsukaguchi, 1995 (Guangxi, Longzhou, female, CAU) **4**Ankylopteryx (A.) gracilis Nakahara, 1955 (Guangxi, Ningming, female, CAU) **5**Ankylopteryx (A.) lii Yang, 1987 (Xizang, Zäyu, paratype, male, CAU) **6**Ankylopteryx (A.) magnimaculata Yang, 1987 (Xizang, Zäyu, holotype, male, CAU) **7**Ankylopteryx (A.) octopunctata candida (Fabricius, 1798) (Guangxi, Ningming, female, CAU) **8**Ankylopteryx (A.) yangi sp. nov. (Taiwan, Pingtung, paratype, female, CAU) **9**Ankylopteryx (A.) tibetana Yang, 1987 (Xizang, Zäyu, holotype, male, CAU). Scale bars: 5.0 mm.

**Figures 10–14.**
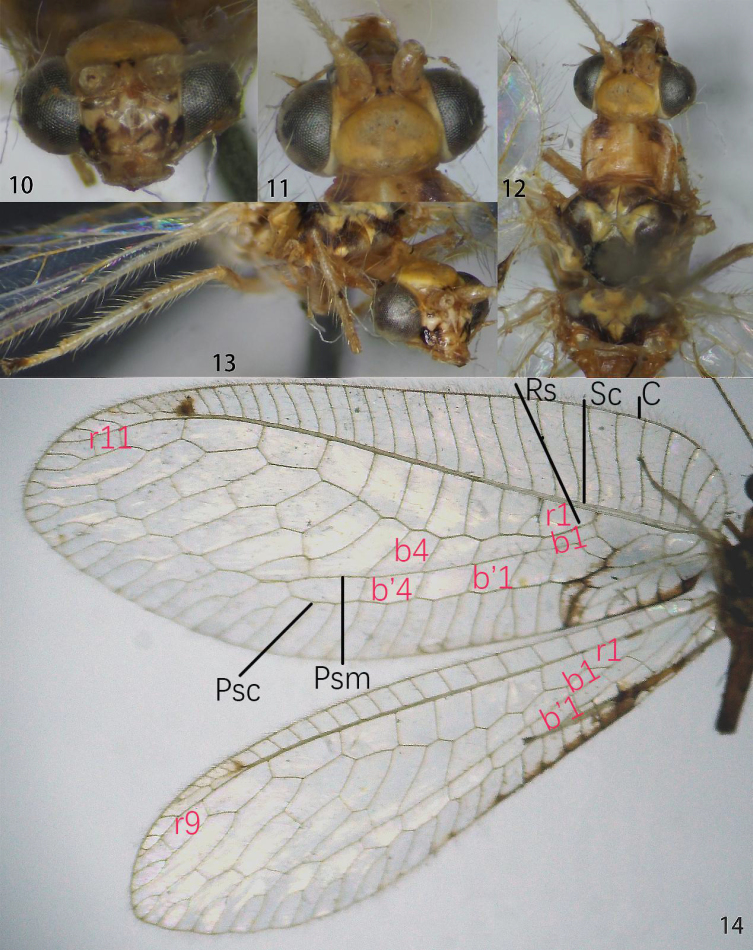
Ankylopteryx (A.) delicatula Banks (Yunnan, Jinghong, sex unknown, CAU). **10** head, frontal **11** head, dorsal **12** thorax, dorsal **13** protibia and mesotibia **14** forewing and hind wing. Veins (black lettering): **C** Costa **Sc** Subcosta **R** Radius **Rs** Radial sector **Psc** Pseudocubitus **Psm** Pseudomedia. Cells (red lettering): **b1, b4** first, fourth upper Banksian cells **b'1, b'4** first, fourth lower Banksian cells **r1, r9, r11** first, ninth, eleventh radial cell.

**Figures 15–17.**
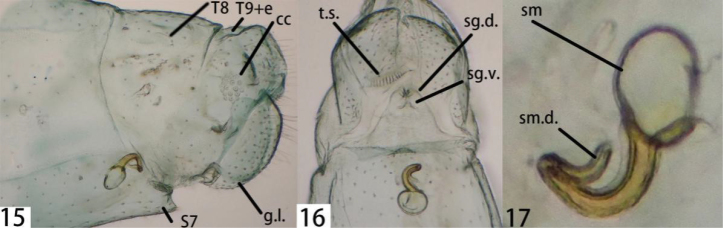
Ankylopteryx (A.) delicatula Banks, female abdomen (Yunnan, Jinghong, sex unknown, CAU). **15** segment A7-terminus, lateral **16** terminalia, ventral **17** spermatheca. **cc** callus cerci **g.l.** gonaphophyses lateralis **S7** seventh sternum **sg.d.** dorsal lobe of subgenitale **sg.v.** ventral lobe of subgenitale **sm** spermatheca **sm.d.** spermathecal duct **t.s.** transverse sclerite **T8** eighth tergum **T9+e** ninth tergum + ectoproct.

**Figures 18–22.**
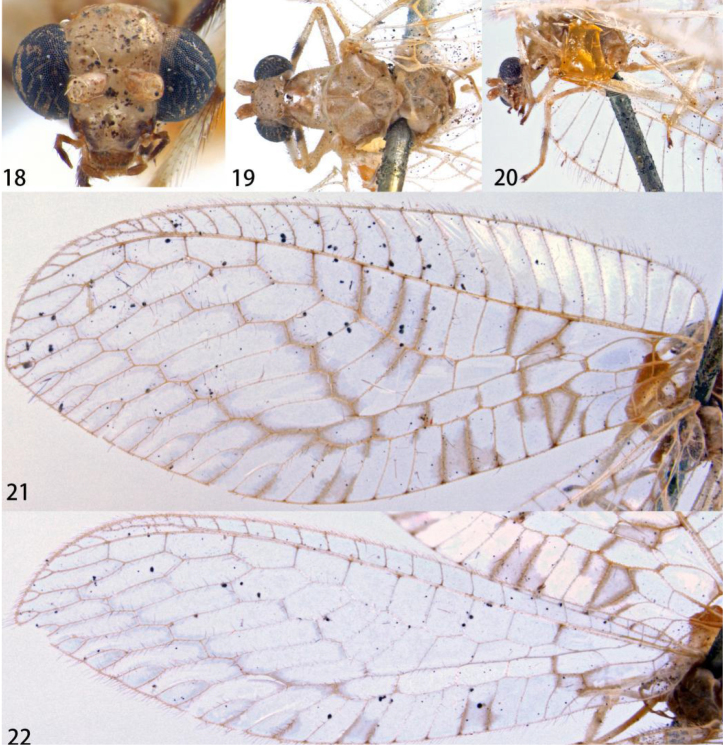
Ankylopteryx (A.) doleschalii Brauer (Indonesia, Ambonia, holotype, ex, provided by NHMV). **18** head, frontal **19** head and thorax, dorsal **20** protibia and mesotibia **21** forewing **22** hind wing.

**Figures 23–29.**
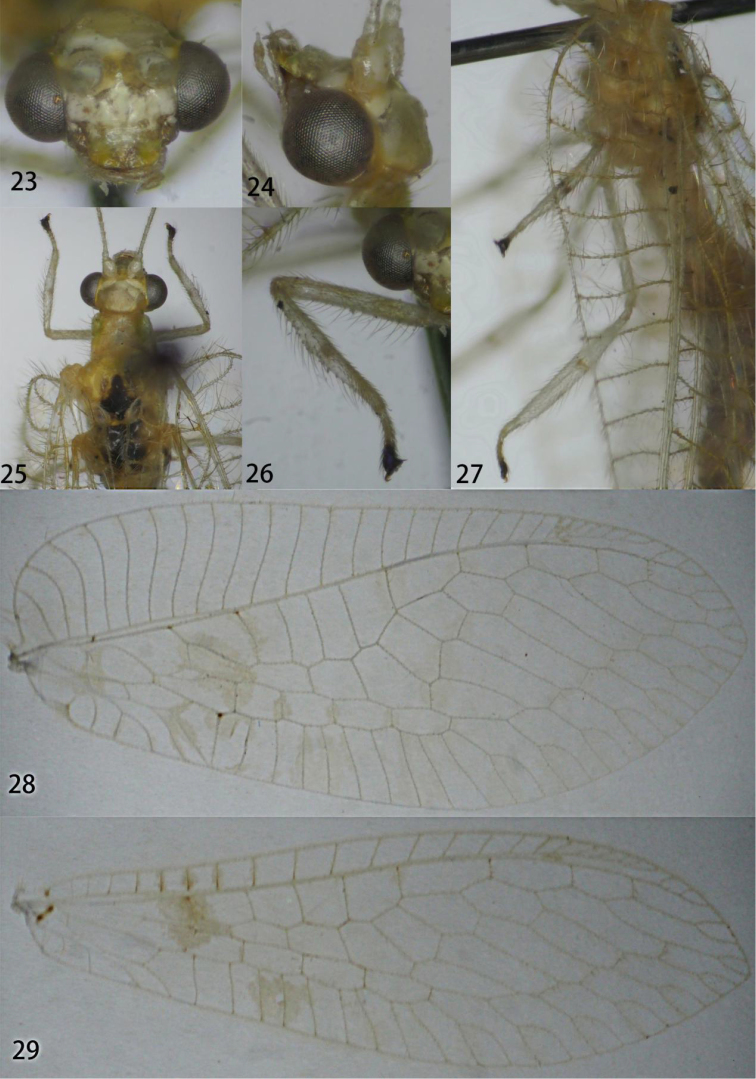
Ankylopteryx (A.) ferruginea Tsukaguchi. **23** head, frontal **24** head, lateral **25** thorax, dorsal **26** protibia **27** mesotibia and metatibia **28** forewing **29** hind wing (23–27: Yunnan, Lancang, male, CAU; 28, 29: Hainan, Yinggeling, female, CAU).

**Figures 30–35.**
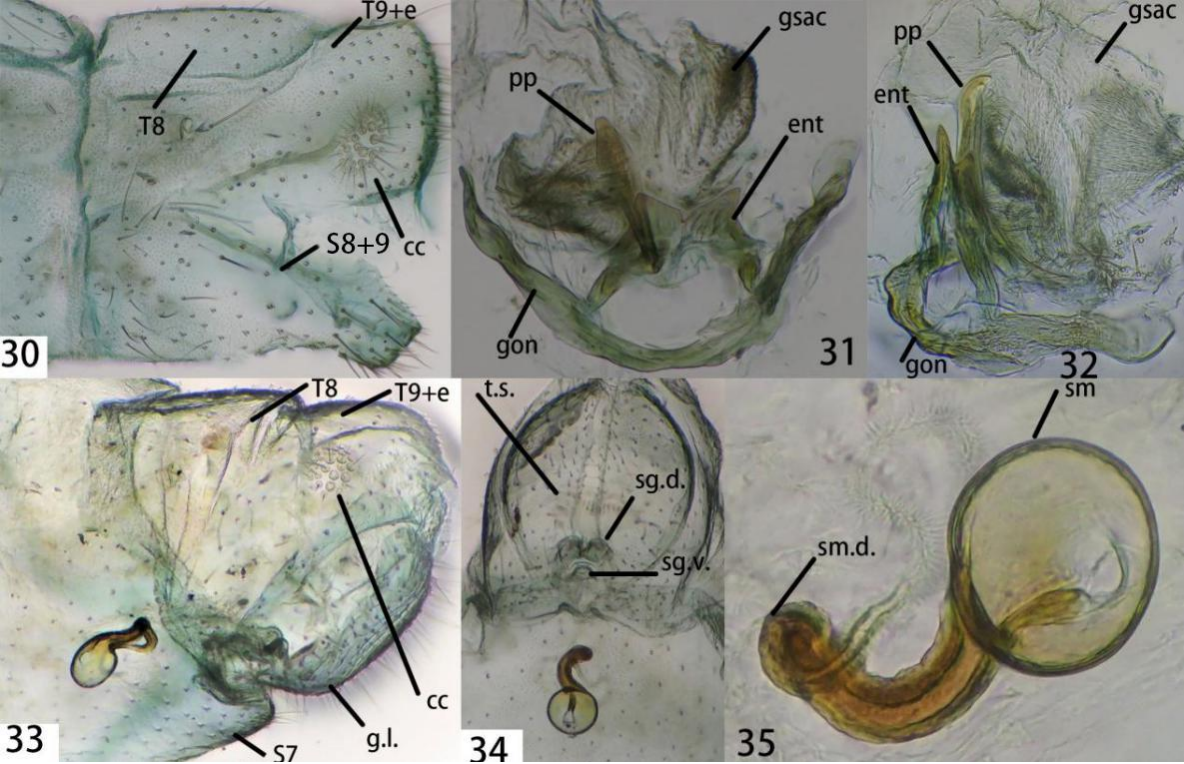
**30–32**Ankylopteryx (A.) ferruginea Tsukaguchi, male abdomen (Yunnan, Lancang, male, CAU). **30** segment A7-terminus, lateral **31** gonarcal complex, dorsal **32** gonarcal complex, lateral. **33–35**Ankylopteryx (A.) ferruginea Tsukaguchi, female abdomen (Hainan, Yinggeling, female, CAU). **33** segment A7-terminus, lateral **34** terminalia, ventral **35** spermatheca. **cc** callus cerci **ent** entoprocessus **g.l.** gonaphophyses lateralis **gsac** gonosaccus **gon** gonarcus **pp** pseudopenis **S7** seventh sternum **S8+9** fused eighth+ ninth sternum **sg.d.** dorsal lobe of subgenitale **sg.v.** ventral lobe of subgenitale **sm** spermatheca **sm.d.** spermathecal duct **t.s.** transverse sclerite **T8** eighth tergum **T9+e** ninth tergum + ectoproct.

**Figures 36–40.**
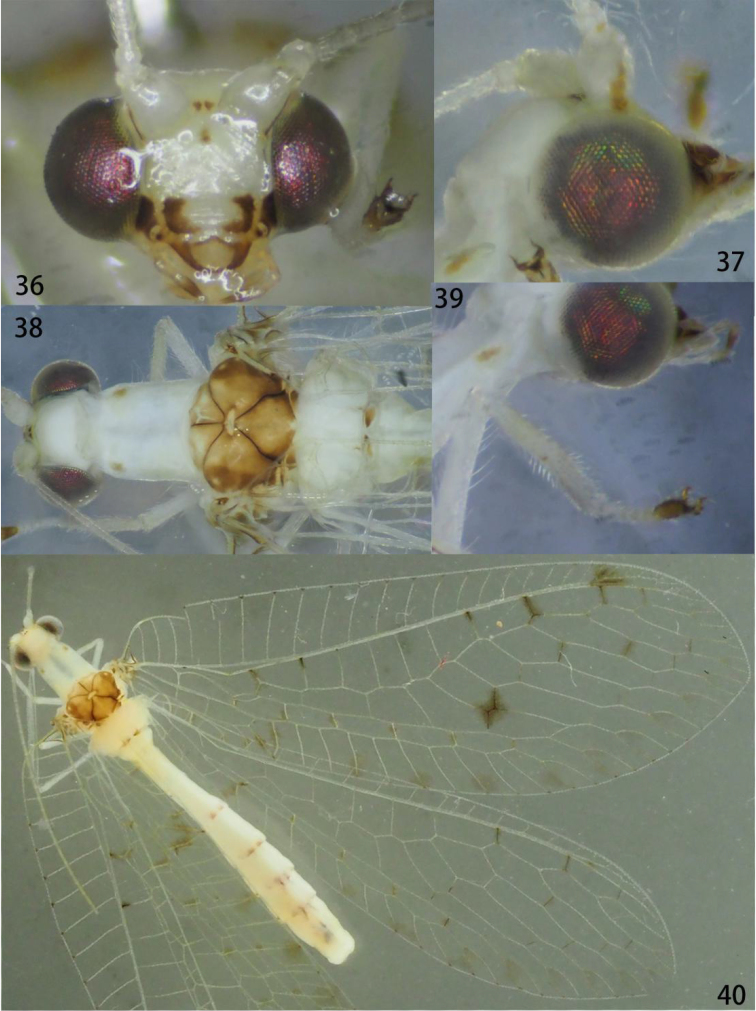
Ankylopteryx (A.) gracilis Nakahara (Japan, Okinawa, Iriomote-jima, male, CAU). **36** head, frontal **37** head, lateral **38** head and thorax, dorsal **39** protibia **40** forewing and hind wing.

**Figures 41–47.**
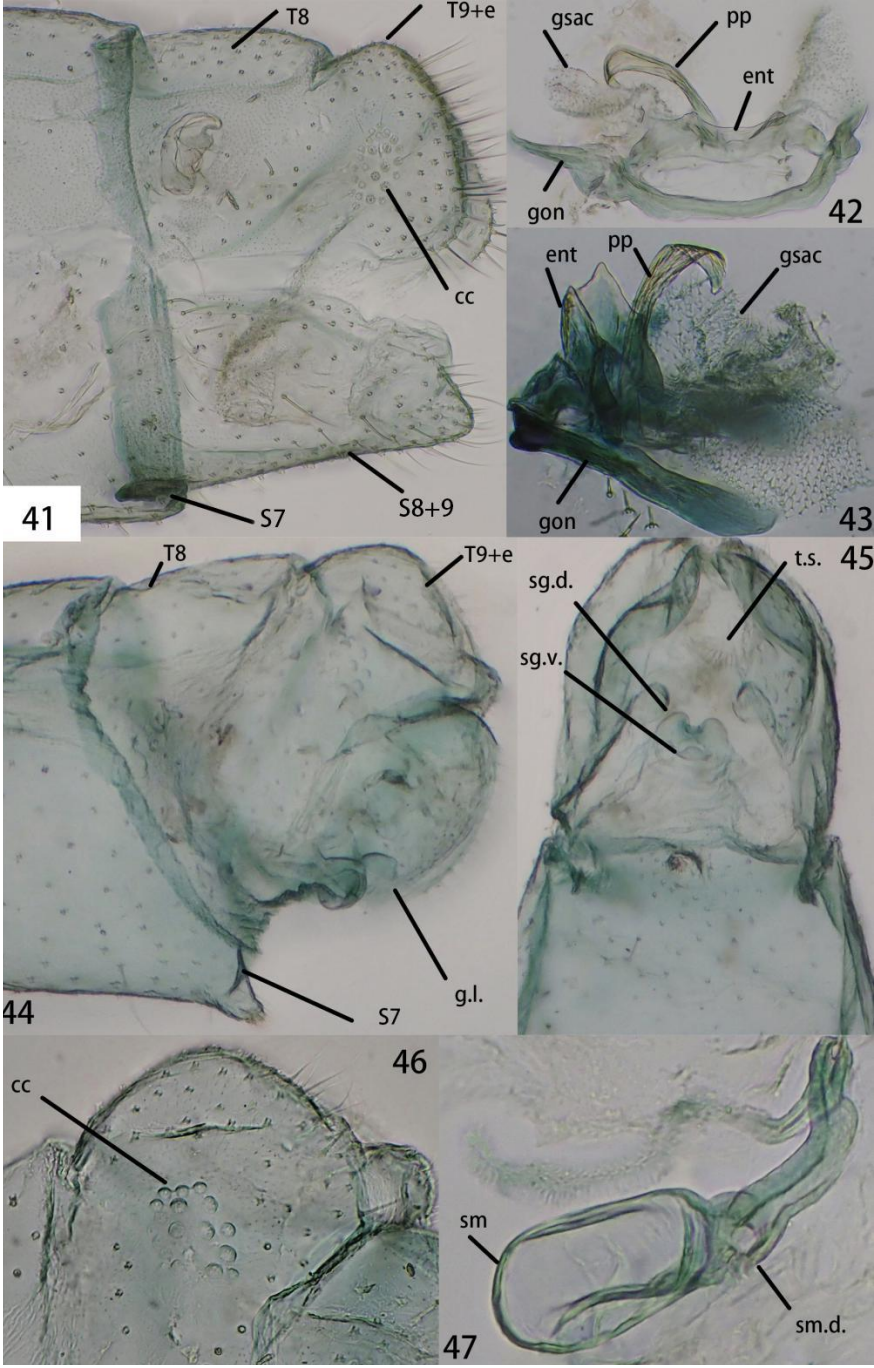
**41–43**Ankylopteryx (A.) gracilis Nakahara, male abdomen (Japan, Okinawa, Iriomote-jima, male, CAU). **41** segment A7-terminus, lateral **42** gonarcal complex, dorsal **43** gonarcal complex, lateral. **44–47**Ankylopteryx (A.) gracilis Nakahara, female abdomen (Guangxi, Ningming, female, CAU). **44** segment A7-terminus, lateral **45** terminalia, ventral **46** callus cerci **47** spermatheca. **cc** callus cerci **ent** entoprocessus **g.l.** gonaphophyses lateralis **gsac** gonosaccus **gon** gonarcus **pp** pseudopenis **S7** seventh sternum **S8+9** fused eighth + ninth sternum **sg.d.** dorsal lobe of subgenitale **sg.v.** ventral lobe of subgenitale **sm** spermatheca **sm.d.** spermathecal duct **t.s.** transverse sclerite **T8** eighth tergum **T9+e** ninth tergum + ectoproct.

**Figures 48–53.**
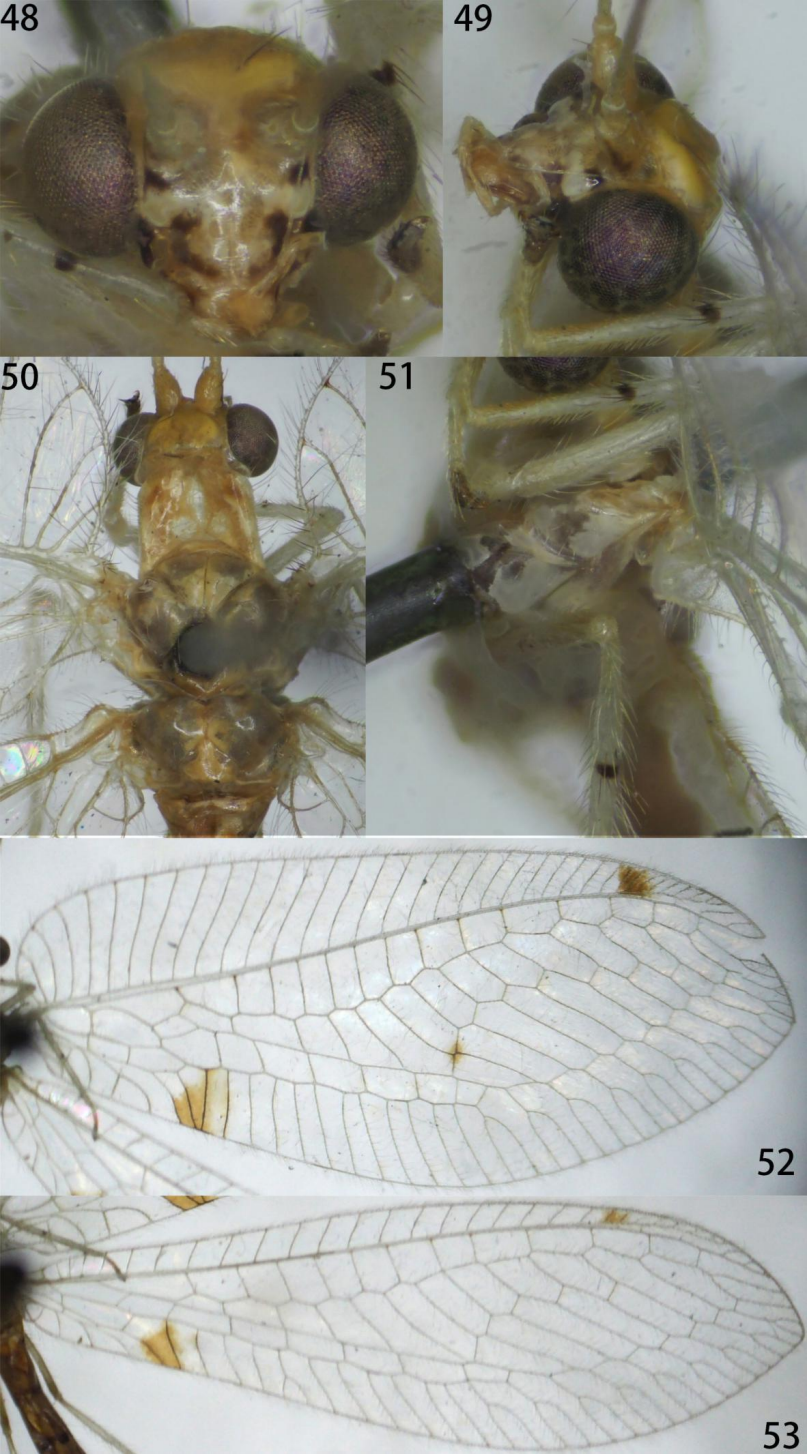
Ankylopteryx (A.) lii Yang (Xizang, Zäyu, holotype, male, CAU). **48** head, frontal **49** head, lateral **50** head and thorax, dorsal **51** protibia and mesotibia **52** forewing **53** hind wing.

**Figures 54–57.**
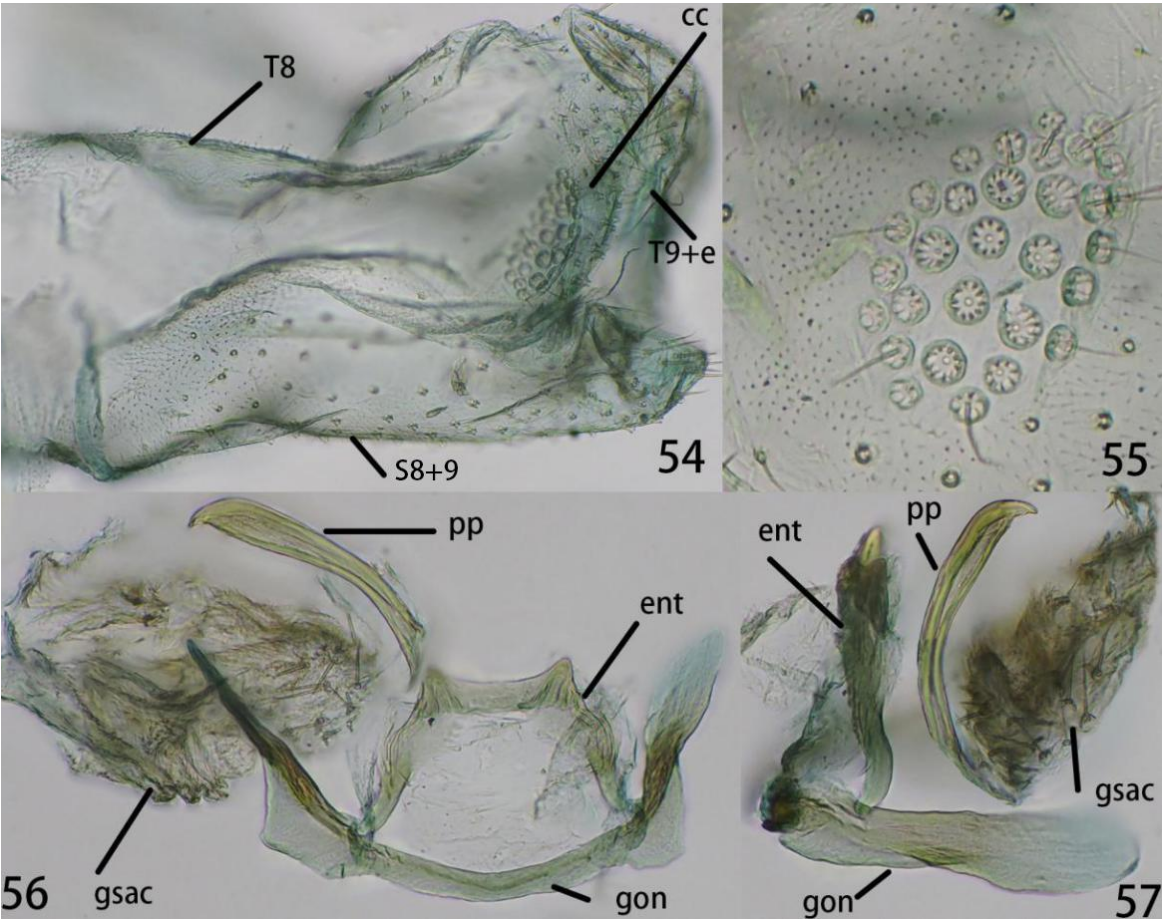
Ankylopteryx (A.) lii Yang, male abdomen (Xizang, Zäyu, holotype, male, CAU). **54** segment A7-terminus, lateral **55** callus cerci **56** gonarcal complex, dorsal **57** gonarcal complex, lateral. **cc** callus cerci **ent** entoprocessus **gsac** gonosaccus **gon** gonarcus **pp** pseudopenis **S8+9** fused eighth + ninth sternum **T8** eighth tergum **T9+e** ninth tergum + ectoproct.

**Figures 58–63.**
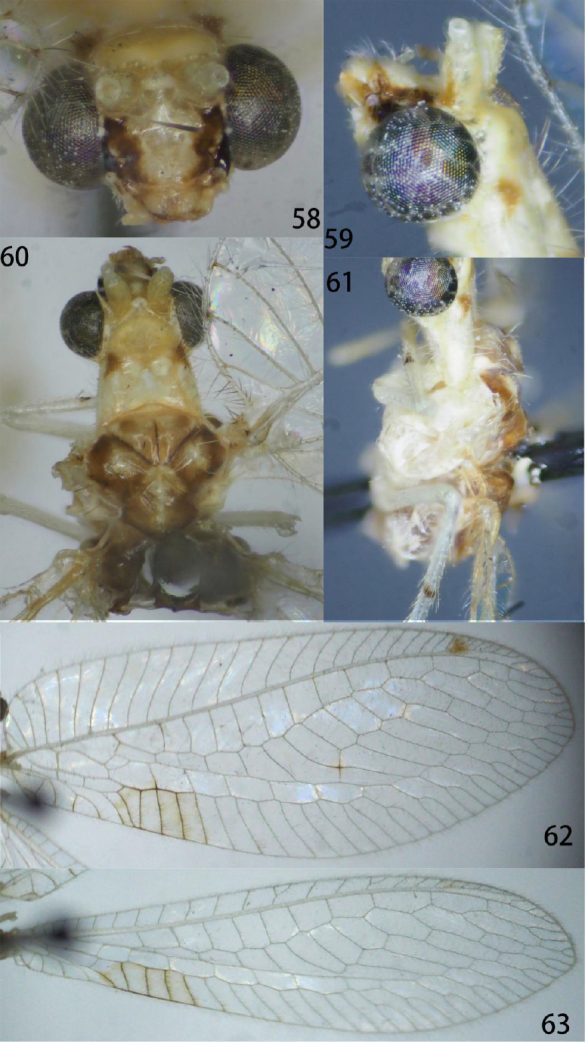
Ankylopteryx (A.) magnimaculatus Yang (Xizang, Zäyu, holotype, male, CAU). **58** head, frontal **59** head, lateral **60** head and thorax, dorsal **61** protibia and mesotibia **62** forewing **63** hind wing.

**Figures 64–66.**
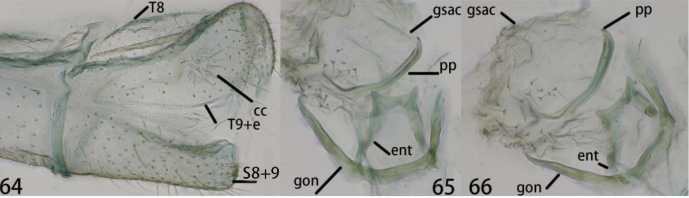
Ankylopteryx (A.) lii Yang, male abdomen (Xizang, Zäyu, holotype, male, CAU). **64** segment A7-terminus, lateral **65** gonarcal complex, dorsal **66** gonarcal complex, dorsolateral. **cc** callus cerci **ent** entoprocessus **gsac** gonosaccus **gon** gonarcus **pp** pseudopenis **S8+9** fused eighth + ninth sternum **T8** eighth tergum **T9+e** ninth tergum + ectoproct.

**Figures 67–72.**
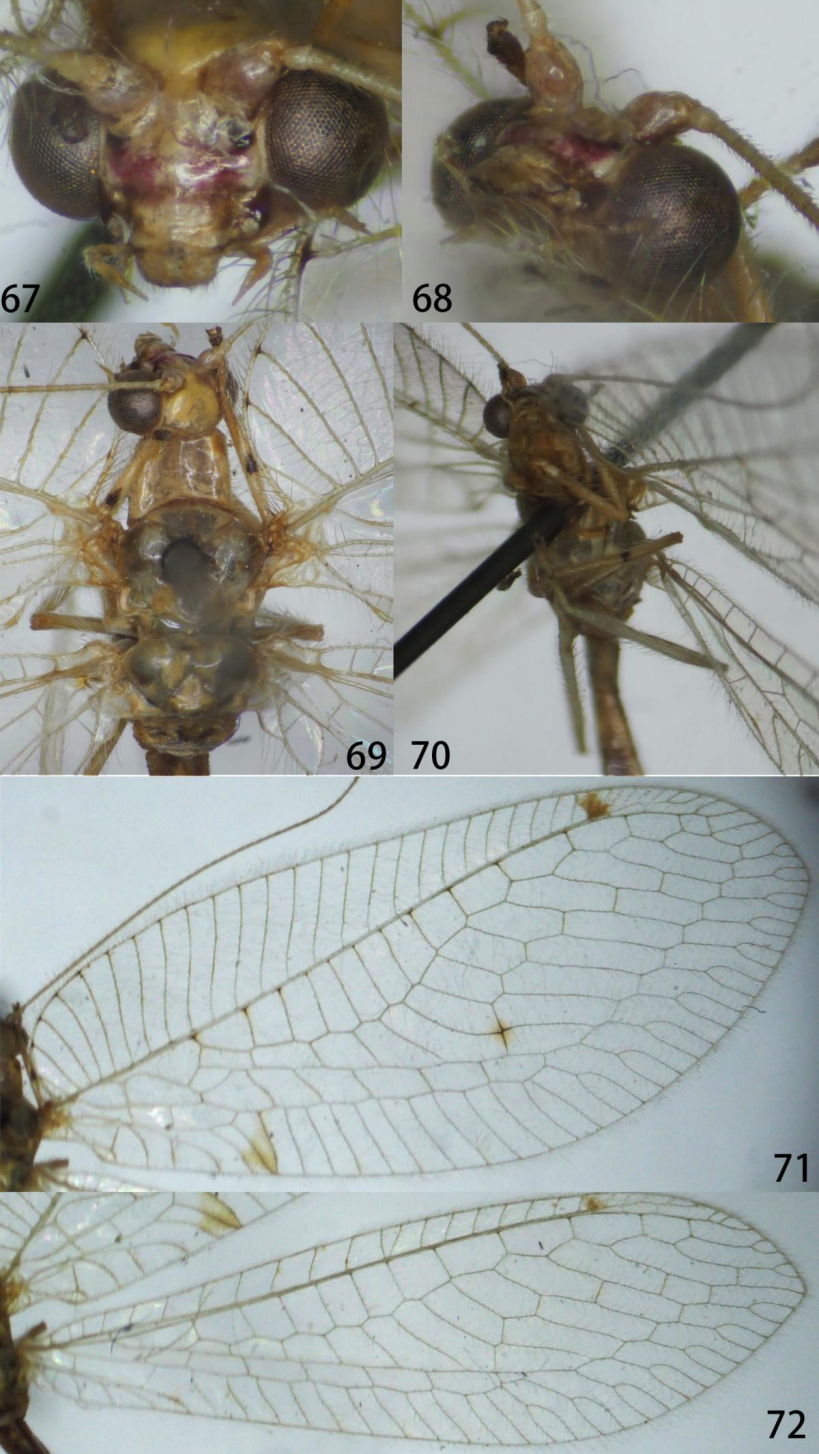
Ankylopteryx (A.) octopunctata candida (Fabricis) (Guangxi, Ningming, male, CAU). **67** head, frontal **68** head, frontolateral **69** head, thorax and protibia, dorsal **70** mesotibia **71** forewing **72** hind wing.

**Figures 73–76.**
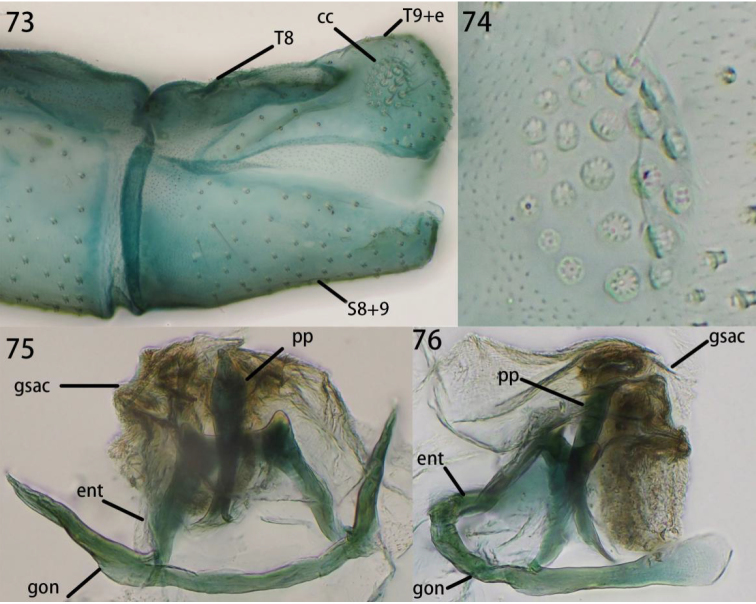
Ankylopteryx (A.) octopunctata candida Fabricius, male abdomen (Laos, Luang Namtha, male, CAU). **73** segment A7-terminus, lateral **74** callus cerci **75** gonarcal complex, dorsal **76** gonarcal complex, lateral. **cc** callus cerci **ent** entoprocessus **gsac** gonosaccus **gon** gonarcus **pp** pseudopenis **S8+9** fused eighth + ninth sternum **T8** eighth tergum **T9+e** ninth tergum + ectoproct.

**Figures 77–82.**
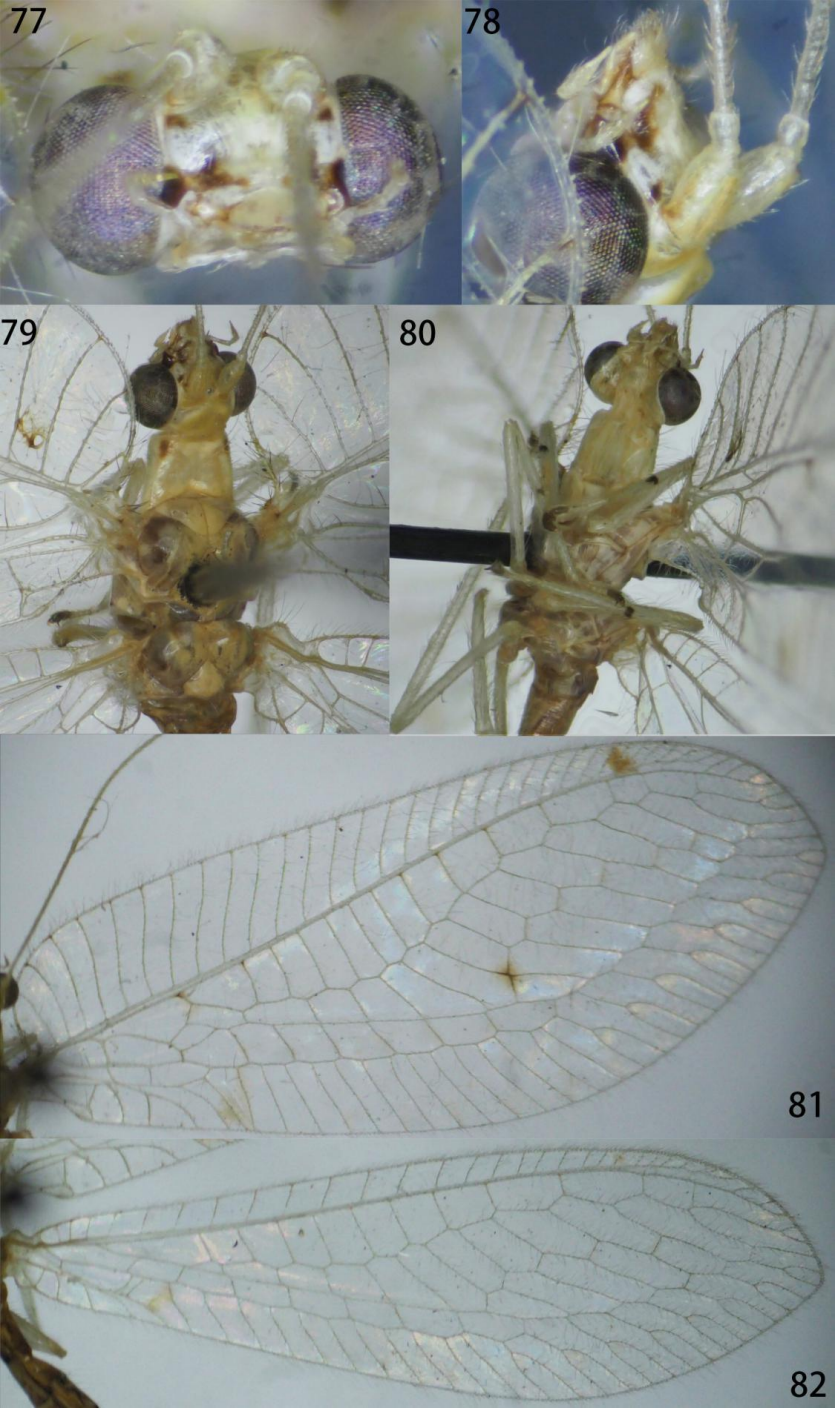
Ankylopteryx (A.) tibetana Yang (Xizang, Zäyu, paratype, male, CAU). **77** head, frontal **78** head, lateral **79** head, thorax and protibia, dorsal **80** protibia and mesotibia **81** forewing **82** hind wing.

**Figures 83–85.**
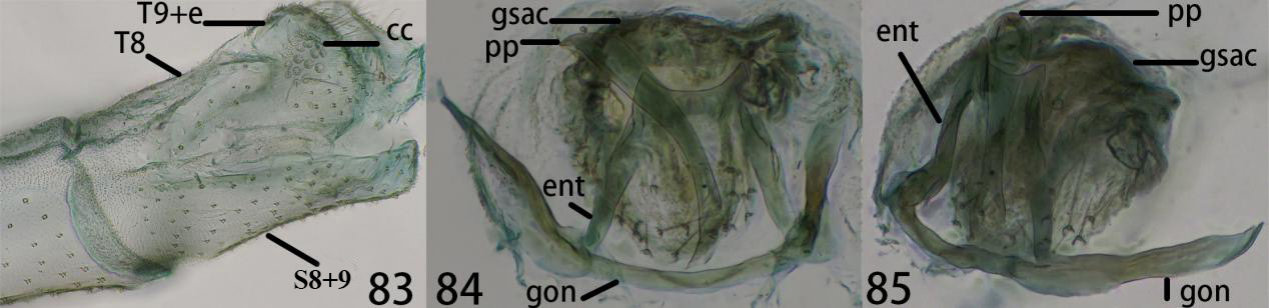
Ankylopteryx (A.) tibetana Yang, male abdomen (Xizang, Zäyu, holotype, male, CAU). **83** segment A7-terminus, lateral **84** callus cerci **85** gonarcal complex, dorsal. **cc** callus cerci **ent** entoprocessus **gsac** gonosaccus **gon** gonarcus **pp** pseudopenis **S8+9** fused eighth + ninth sternum **T8** eighth tergum **T9+e** ninth tergum + ectoproct.

**Figures 86–90.**
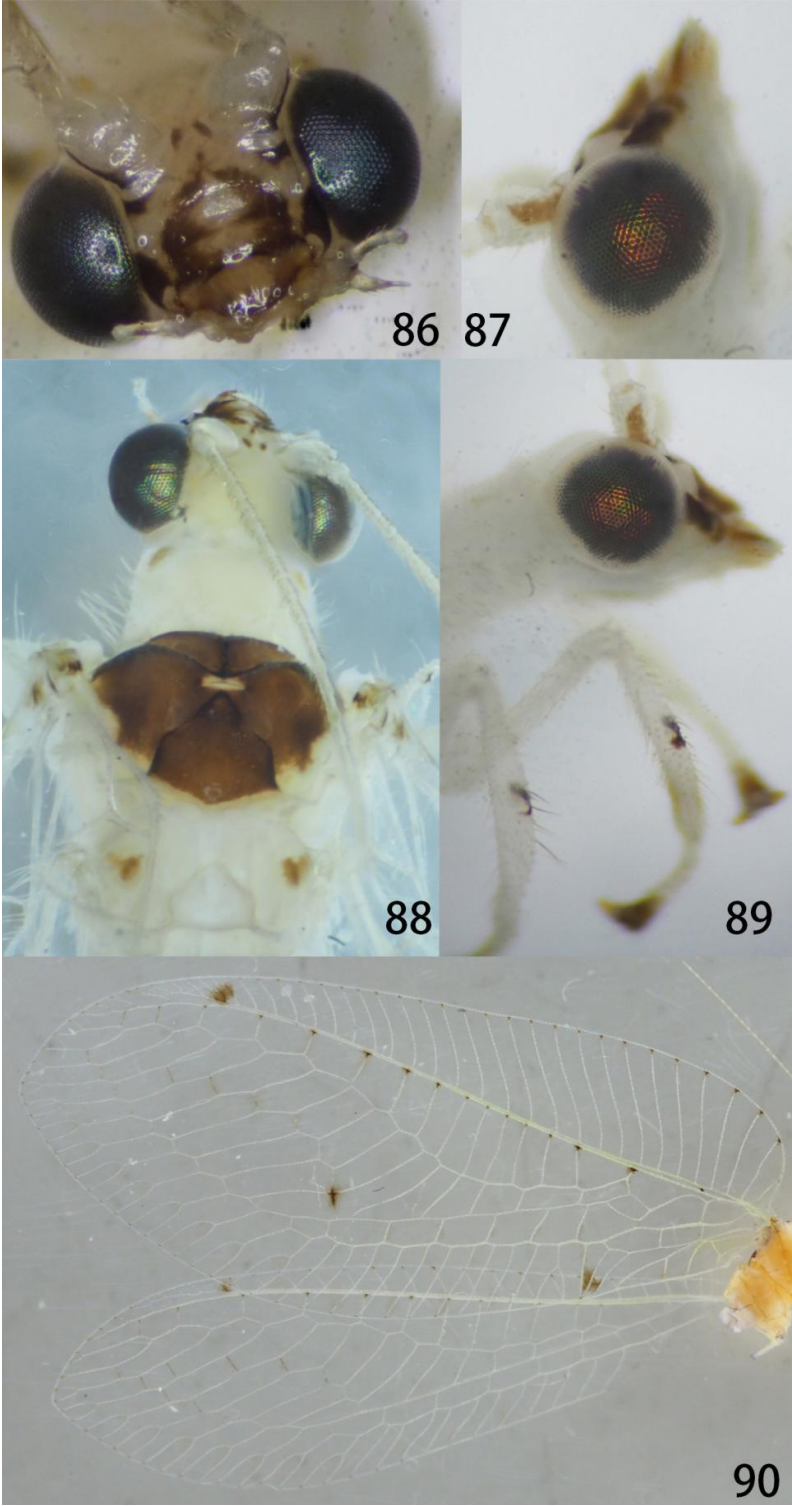
Ankylopteryx (A.) yangi sp. nov. (Guizhou, Libo, holotype, male, CAU). **86** head, frontal **87** head, lateral **88** head and thorax, dorsal **89** protibia and mesotibia **90** forewing and hind wing.

**Figures 91–98.**
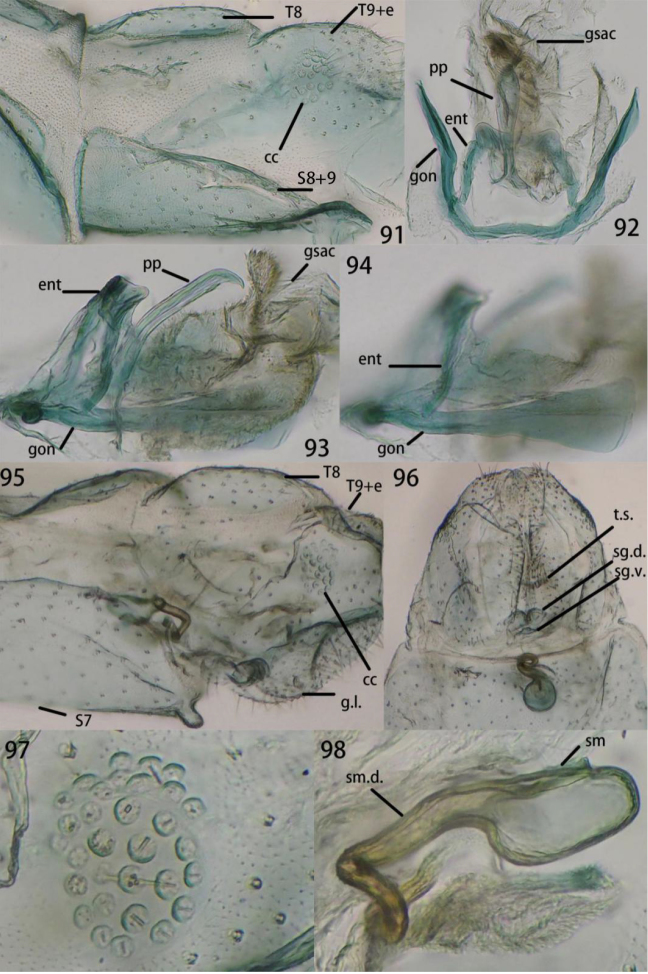
**91–94**Ankylopteryx (A.) yangi sp. nov., male abdomen (Guizhou, Libo, holotype, male, CAU). **91** segment A7-terminus, lateral **92** gonarcal complex, dorsal **93** gonarcal complex, lateral **94** gonarcus. **95–98**Ankylopteryx (A.) yangi sp. nov., female abdomen (Guizhou, Libo, paratype, female, CAU). **95** segment A7-terminus, lateral **96** terminalia, ventral **97** callus cerci **98** spermatheca. **cc** callus cerci **ent** entoprocessus **g.l.** gonaphophyses lateralis **gsac** gonosaccus **gon** gonarcus **pp** pseudopenis **S7** seventh sternum **S8+9** fused eighth + ninth sternum **sg.d.** dorsal lobe of subgenitale **sg.v.** ventral lobe of subgenitale **sm** spermatheca **sm.d.** spermathecal duct **t.s.** transverse sclerite **T8** eighth tergum **T9+e** ninth tergum + ectoproct.

**Figures 99–107.**
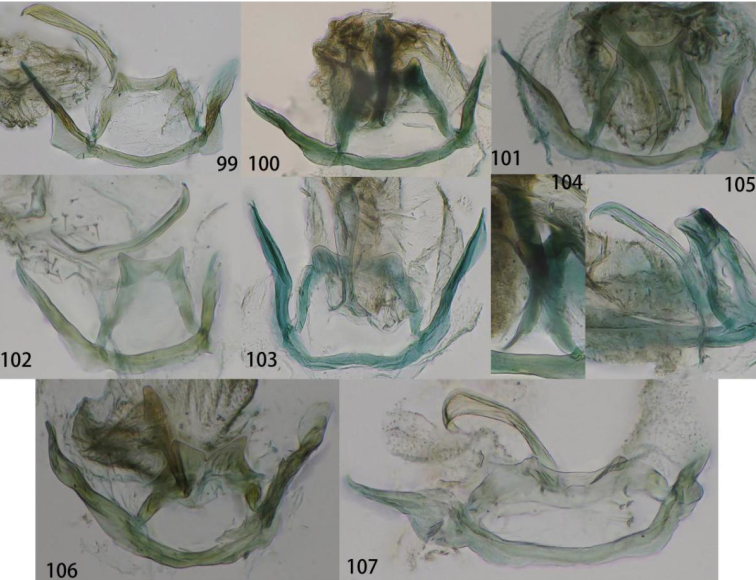
Gonarcal complex. **99**Ankylopteryx (A.) lii Yang, dorsal **100**Ankylopteryx (A.) octopunctata candida (Fabricius), dorsal **101**Ankylopteryx (A.) tibetana Yang, dorsal **102**Ankylopteryx (A.) magnimaculatus Yang, dorsal **103**Ankylopteryx (A.) yangi sp. nov., dorsal **104**Ankylopteryx (A.) octopunctata candida (Fabricius), lateral **105**Ankylopteryx (A.) yangi sp. nov., lateral **106**Ankylopteryx (A.) ferruginea Tsukaguchi, dorsal **107**Ankylopteryx (A.) gracilis Nakahara, dorsal.

**Figures 108–111.**
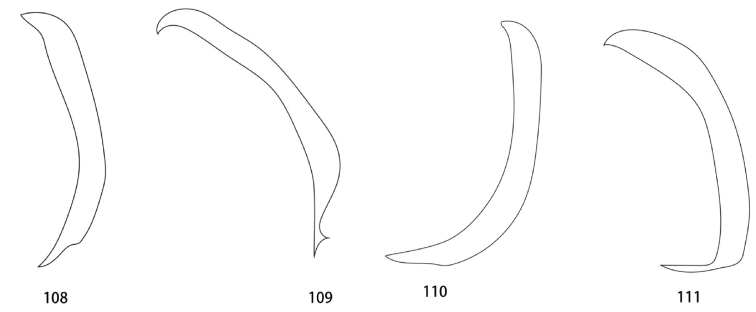
Pseudopenis, line drawings in lateral view. **108**Ankylopteryx (A.) octopunctata candida (Fabricius) **109**Ankylopteryx (A.) yangi sp. nov. **110**Ankylopteryx (A.) ferruginea Tsukaguchi **111**Ankylopteryx (A.) gracilis Nakahara

**Figure 112.**
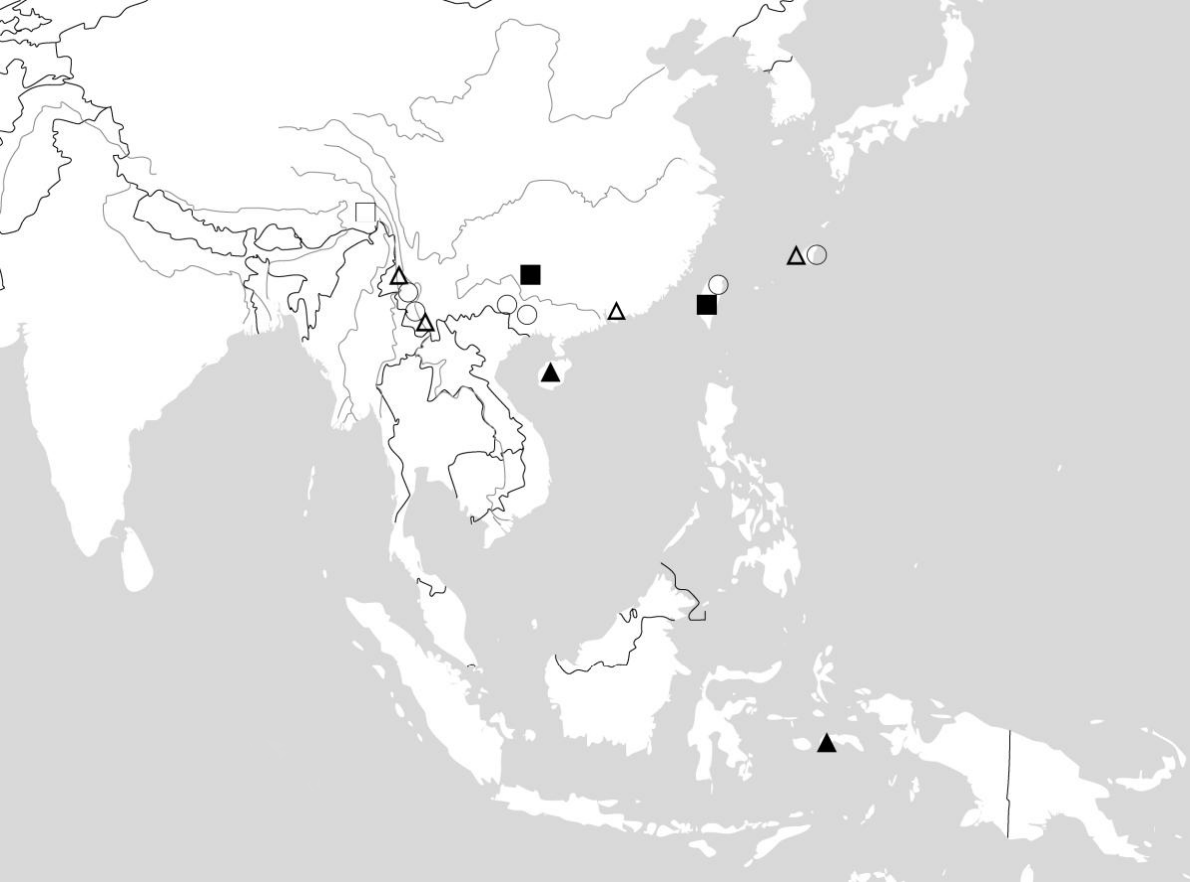
Known distribution of *Ankylopteryx* (*s. str.*) species from China and neighboring countries. Ankylopteryx (A.) delicatula Banks (white triangle); Ankylopteryx (A.) doleschalii Brauer (black triangle); Ankylopteryx (A.) ferruginea Tsukaguchi (circle); Ankylopteryx (A.) magnimaculata Yang (white square); Ankylopteryx (A.) yangi sp. n. (black square).

**Figure 113.**
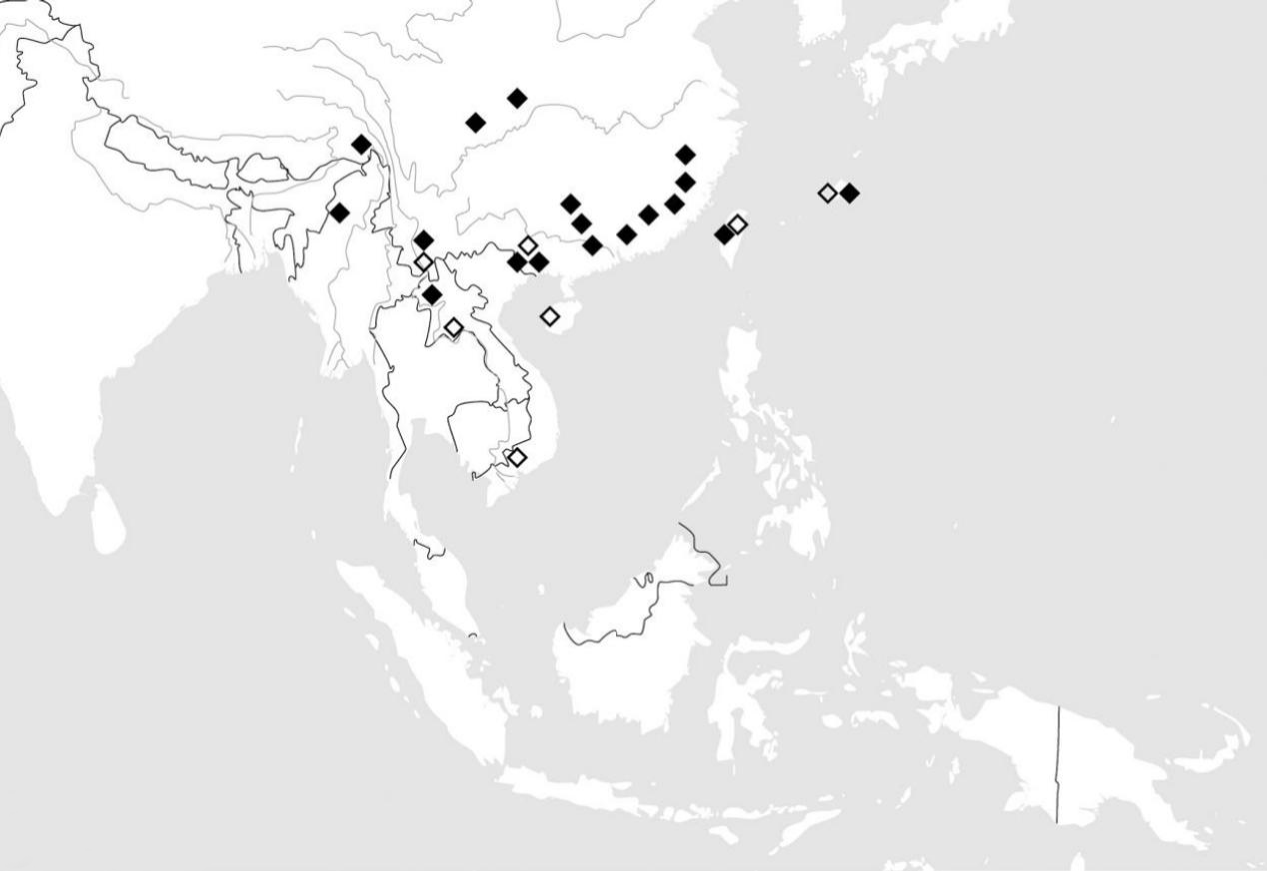
Known distribution of *Ankylopteryx* (*s. str.*) species from China and neighboring countries. Ankylopteryx (A.) gracilis Nakahara (white diamond); Ankylopteryx (A.) octopunctata candida Fabricius (black diamond).

## Supplementary Material

XML Treatment for Subgenus Ankylopteryx

XML Treatment for Ankylopteryx (A.) delicatula

XML Treatment for Ankylopteryx (A.) doleschalii

XML Treatment for Ankylopteryx (A.) ferruginea

XML Treatment for Ankylopteryx (A.) gracilis

XML Treatment for Ankylopteryx (A.) magnimaculata

XML Treatment for Ankylopteryx (A.) octopunctatacandida

XML Treatment for Ankylopteryx (A.) quadrimaculata

XML Treatment for Ankylopteryx (A.) yangi

## Footnotes

A. quadrimaculata is not included in the key. See Remarks for this species.
